# Transforming the Health Research Workforce in Mozambique: Achievements of the Mozambique Institute for Health Education and Research (MIHER) over a 13‑Year Journey

**DOI:** 10.5334/aogh.4528

**Published:** 2024-12-09

**Authors:** Emília Virgínia Noormahomed, Sérgio Noormahomed, Matchecane Cossa, Nicole Joyce, Regina Daniel Miambo, Irina Mendes Sousa, Noémia Nhacupe, Tufária Mussá, Jahit Sacarlal, Lídia Gouveia, Luís Jorge Ferrão, Carla Carrilho, Mamudo Ismail, Davey Smith, Natasha K. Martin, Ravi Goyal, Kim E. Barrett, Sónia Santana Afonso, Amélia Mandane, Alarquia Saíde, Pompílio Vintuar, Brígida Singo, Boaventura Aleixo, Luck Injage, Elizabeth A. Winzeler, Paulo Correia-de-Sá, Maria do Rosário Oliveira Martins, Paulo Ferrinho, Sam Patel, Ana Olga Mocumbi, Stephen W. Bickler, Constance A. Benson, Roberto Badaró, Robert T. Schooley

**Affiliations:** 1 Eduardo Mondlane University (UEM), Maputo, Mozambique; 2 University of California San Diego (UCSD), United States of America; 3 Mozambique Institute for Health Education and Research (MIHER), Maputo, Mozambique; 4 Ministry of Health of Mozambique, Mozambique; 5 Maputo Central Hospital, Maputo, Mozambique; 6 Maputo Pedagogic University, Maputo, Mozambique; 7 School of Medicine, University of California, Davis, Sacramento, California, United States of America; 8 Lúrio University, Nampula, Mozambique; 9 Púngué University, Nampula, Mozambique; 10 Licungo University, Zambézia, Mozambique; 11 Instituto de Ciências Biomédicas Abel Salazar, Universidade do Porto (ICBAS‑UP), Porto, Portugal; 12 Global Health and Tropical Medicine (GHTM), Associate Laboratory in Translation and Innovation Towards Global Health (LA‑REAL), Instituto de Higiene e Medicina Tropical (IHMT), Universidade NOVA de Lisboa (UNL), Lisbon, Portugal; 13 Instituto Nacional de Saúde, Maputo, Mozambique; 14 Federal University of Bahia (UFBA), Salvador, Brazil

**Keywords:** Mozambique Institute for Health Education and Research (MIHER), MIHER, Research Support Center (RSC), Research capacity development, Medical Education Partnership Initiative (MEPI), Health Professional Education Partnership Initiative (HEPI), Research Administration, Health Professionals Education, Mozambique

## Abstract

*Background:* African research capacity is challenged by insufficient infrastructure to solicit and manage grants from local and international funding agencies.

*Objective:* The manuscript provides an overview and discusses lessons learned about the pioneering role of the Mozambique Institute for Health Education and Research (MIHER) as the first research support center (RSC) in supporting the management of research grants in Mozambique, emphasizing its impact on research capacity development.

*Methods:* Using mixed methods, data were comprehensively collected to identify MIHER’s primary achievements from 2010 to 2023. The activities took place in four public universities, five training institutions for healthcare workers, and 40 public healthcare units in Mozambique.

*Findings:* MIHER had partnership contracts with over 35 external institutions, and supported the design and implementation of one doctoral program and five masters’ degree programs at three public universities. Over 70% of the 128 MSc and three Ph.D. degree recipients have gone on to become lecturers at Mozambique’s public universities or are working in Mozambique’s public health system. Over 9,000 lecturers and healthcare workers participated in MIHER’s 261 research capacity development workshops. MIHER assisted in writing and implementing 98 research grants, amassing $29,923,197 in extramural support. Of 170 publications generated, 89% were indexed in PubMed. African researchers served as first or last author in 55% and 34% of these publications, respectively; Mozambicans were first and last authors in 44% and 23% of the articles, respectively. Two research laboratories were rehabilitated. Investments in information and communication technology also fostered training and mentorship.

*Conclusions:* MIHER has emerged as a leading RSC of Excellence, fostering synergies and promoting a quality research culture in Mozambique, fueled in part, by its ability to identify and incorporate key collaborations. MIHER is a successful example of an RSC that can make the difference in resource‑limited settings to enable research resource mobilization, evidence-based health care delivery and policy design.

## Background

Mozambique, a Portuguese‑speaking, low‑income African country, is challenged by significant development issues, including being one of the African countries most prone to extreme weather events [1, 2]. Mozambique ranks 183 out of 193 countries in the Human Development Index [3]. Furthermore, it has one of Africa’s lowest per capita rates of higher education participation. From 2004 to 2010, few Mozambican university lecturers had a master’s (22%) or Ph.D. (11%) degree, and most of those qualifications were obtained abroad. This education deficit has also impacted the size of the country’s healthcare workforce, with one of the most critical shortages in the world that is complicated by an uneven geographic distribution of resources, felt particularly in the country’s north and central regions. These constraints add multiple layers of challenge in a country with a high burden of malnutrition and communicable and non‑communicable diseases [4].

### Higher education institutions involved in the development of healthcare system workers

Created in 1963 during the Portuguese colonization, the country’s national flagship university, Eduardo Mondlane University (UEM), is located in Mozambique’s capital city, Maputo. It remained the only public university training physicians for the entire country during the first 41 years after the 1975 independence from Portuguese rule [2, 5].

Mid‑level nurses and other healthcare workers (HCW) received training in schools across the country under the leadership of the Ministry of Health (MOH). As part of the Mozambique governmental strategy for extension of higher education and research development, higher education was expanded to other geographical regions in the country [2].

With 10 branches across Mozambique’s 10 provinces, the Universidade Pedagógica de Moçambique (UPM) was initially created in 1985 to train teachers for the primary and secondary education system [2]. In 2019, UPM was restructured, and the Licungo University (UniLicungo) was created in central Mozambique, with Mozambique’s newest Faculty of Medicine [6].

The Instituto Superior de Ciências de Saúde (ISCISA) was founded in 2003, with two main campuses. One is located in Maputo, and the other in Quelimane, a city located in the Zambeze province in central Mozambique, to support advanced training of mid‑level HCW (nurses, non‑physician technicians) previously trained under the umbrella of the MOH [7].

Lurio University (UniLurio) and Zambeze University (UniZambeze) were created in 2006 and 2007, respectively, based in geographically underserved but highly populated provinces of northern (Nampula) and central (Zambeze) Mozambique, respectively. Each includes a Faculty of Health Sciences to train physicians and other HCW [2].

Private institutions also train HCW. These include the Catholic University (Beira, central Mozambique, created in 1995), and the Instituto Superior de Ciências e Tecnologia de Moçambique in Maputo, created in 1997 [5].

### The medical education partnership initiative

In 2010, through a partnership formed in 2008 between UEM and University of California San Diego (UCSD), UEM received 1 out of 13 Medical Education Partnership Initiative (MEPI) awards from the US President’s Emergency Plan for Acquired Immunodeficiency Syndrome Relief (PEPFAR) and the Fogarty International Center (FIC) of the National Institutes of Health (NIH) of the USA. These awards aimed to increase the number of physicians as critical human resources needed to address human immunodeficiency virus (HIV)/acquired immunodeficiency syndrome (AIDS) control, foster research development within the country by addressing the most pressing health issues, create communities of practice, promote the internationalization of Mozambican universities, and support the development of newly established universities in the country (e.g., UniLurio and UniZambeze), including the creation of research support centers (RSCs) [2, 8–10].

To understand the hurdles and needs to create a robust training and research environment in Mozambique, the MEPI team of investigators, comprising partners from UEM, UniLurio, and UCSD, sought advice from a broad set of key leader institutions, including the MOH, Ministry of Education, Ministry of Science and Technology, Mozambican Medical Council, and UCSD [8].

We also engaged additional universities from Portugal (Universidade NOVA de Lisboa through the Instituto de Higiene e Medicina Tropical, and Universidade do Porto (UP) via the Instituto de Ciências Biomédicas Abel Salazar), as well as from Brazil (Federal University of Bahia) [2, 8].

Major hurdles and weaknesses were identified on the basis of these discussions and of needs assessment. The main barriers included excessively bureaucratic institutional environments, limitations in qualified human resources and research infrastructures, a lack of financial resources, inadequate administrative support for grants, administrative management policies, and weak regulatory structures. Additionally, many investigators had limited access to scientific literature and lacked English proficiency [8].

Based on a multifaceted plan designed to reduce the impact of these barriers and to enhance HCW research capacity in Mozambique, the Mozambique Institute for Health Education and Research (MIHER) was conceived. This was to be a para‑University not‑for‑profit organization conceived in 2010, that was formally instituted in 2011, as the first RSC in Mozambique [8]. Its creation as a not‑for‑profit organization was based on the premise that, to adequately perform their roles, RSCs need to have three key characteristics, namely local ownership, independence, and coordination abilities [11].

Nevertheless, even at the beginning, MEPI‑Mozambique was a consortium of three universities—UEM, UniLurio, and UniZambeze—and aimed also to expand its future activities across the country [8]. MIHER was conceived as the institutional instrument to promote such expansion. MIHER’s major goals were to provide responsive and transparent administrative support, conduct fiscal and project management services for Mozambique’s health and social sciences research communities, and identify funding agencies interested in supporting research and training activities in Mozambique’s public universities training HCW [8].

### Methods

In this case study, we report on the process of creation of MIHER, as well as its main achievements and effect on developing, implementing and sustaining research capacity‑development activities since its creation in 2011, i.e., 13 years ago. We outline the challenges and lessons learned, and how MIHER is contributing to the transformation of the health research workforce (HRW) landscape in Mozambique, as an example for other African countries.

To get a comprehensive picture of the extent of MIHER’s activities, we extracted data from MIHER’s publications (in PubMed, Scopus, and Google Scholar), project progress reports, and activity records, thus offering valuable qualitative and quantitative contributions for analysis through December 15, 2023.

We estimated the following indicators: (a) postgraduate (master and doctoral) programs created and supported, (b) students trained, (c) students sponsored, (d) micro‑research projects mentored, (e) research methods’ courses designed and/or implemented, (f) participants in these courses, (g) research grants written and funded, and (h) publications generated.

Quantitative data such as the number of publications and citations as well as project applications versus those funded were entered into a Microsoft Excel spreadsheet, and exported to the Statistical Package for the Social Sciences (SPSS). Data analysis includes descriptive statistics, tables, and bar and line charts.

The publications retrieved were classified into three groups on the basis of the Scimago Journal & Country Rank (SJR) indicator: (a) publications in high‑citation‑potential journals with a high SJR indicator value (SJR > 1.0), (b) publications in journals with below‑average citation potential (those with an SJR indicator value < 1.0), and (c) publications in non‑SJR indexed journals.

## Case Presentation

### MIHER and its organizational structure

MIHER was initially registered as the “Instituto Moçambicano de Assistência e Apoio à Pesquisa e Ensino em Saúde (IMAPES)” at the Office for Registration of Legal Entities (Conservatória de Entidades Legais) under the authority of Mozambique Ministry of Justice. This was done after fulfilling requirements defined by Mozambican law, which include presentation of the identity of funding members, proof of no criminal record, and the statutes that would guide the organization. In addition, there was a need also to include the composition of MIHER’s social governance bodies, who were elected during an initial General Assembly.

According to MIHER’s statutes, it is mandatory that social governance bodies are chosen from within MIHER’s members [12]. In the same year, IMAPES was renamed as the Mozambique Institute for Health Education and Research (MIHER) to convey its name in English [13]. MIHER’s governance structures initially included 11 MEPI investigators from UEM and Maputo Central Hospital (MCH; 5), MOH (1), Institute Nacional de Saúde (INS) (1), and civil society organizations (CSO) (4). Over the years, other members were admitted, now encompassing 24 members, including honorary members from UCSD (2). MIHER’s General Assembly serves as the highest authority and provides an overarching strategy for the organization’s development. It meets yearly to oversee implementation of activities defined under the institution’s strategic plans and also convenes extra sessions to decide on time‑sensitive issues whenever necessary. The board of directors—with a president, vice president, and general secretary—is responsible for strategic planning and oversight of MIHER´s activities, including seeking any new partnerships. MIHER’s everyday activities are run under the leadership of the executive director. There are two functional units: Research and Training, and Administration. In addition, MIHER has a Fiscal Council of three members: the chair, a secretary, and a third member without a specific portfolio. The Fiscal Council is a supervisory body, independent of the Board of Directors (but also elected by the General Assembly), which seeks, through the principles of transparency, equity, and accountability, to contribute to the organization’s efficient performance.

**Figure 1:**
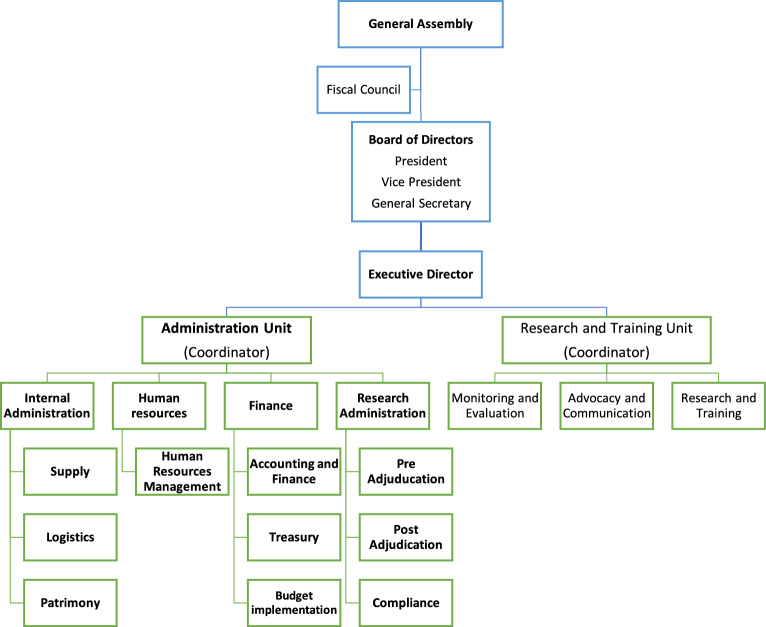
MIHER’s organizational chart.

The organizational structure of MIHER is illustrated in Figure 1. One important feature of the structure—which contrasts with other Mozambican public universities and research institutions—is that the Administration Unit houses the Research Administration Department, which consists of three sub‑units: Pre‑adjudication, Post‑adjudication, and Compliance. The Research and Training Unit plans research and capacity development activities, including advocacy, communication, education, and institutional review board (IRB) compliance.

In terms of human resources, MIHER has 14 full‑time employees engaged in administrative work. Most of its affiliated researchers (approximately 80) are lecturers and health workers from different Mozambican Public Universities, research institutions, and MOH health units.

The MIHER annual budget is approximately $130,000 USD and derives from the administrative and overhead costs of the projects it administers, from donations, and from funds earned by the provision of services (support designing, submission, implementation, and administration of training and research grants and provision of didactic and research method courses and grant and manuscript writing, among others).

### Implementation sites and approach

**Figure 2:**
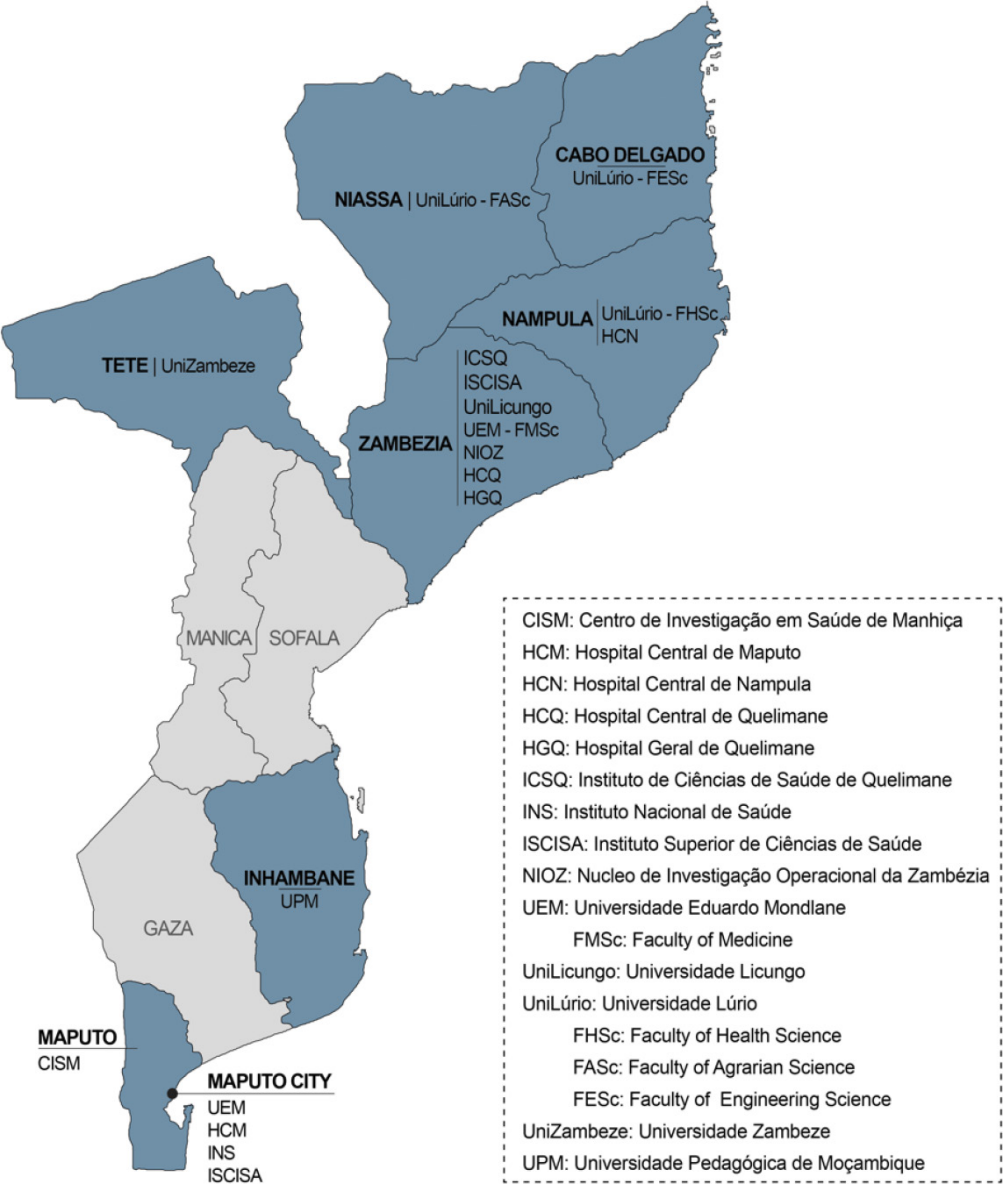
Map highlighting locations of MIHER’s on‑site activities.

On‑site activities took place in only 7 of 10 Mozambican provinces (Figure 2). However, activities conducted using information and communication technology (ICT) tools reached lecturers, researchers, and HCW throughout the country. All public entities training HCW were involved, including Mozambique’s public universities and other higher education institutions, as well as 40 healthcare district and central hospitals.

### Design and implementation of graduate programs and faculty development

Altogether, MIHER supported the design and implementation of three masters’ programs at UniLurio: Health Professionals Education, Tropical Medicine and Global Health, and Nutrition and Food Security [4]. At the time of writing, more than 128 master’s students have graduated since implementation in 2013. At least 70% of the students were faculty members in one of Mozambique’s public universities or HCW in other public institutions, including healthcare facilities.

At UEM, MIHER supported the design and implementation of the first master’s program in Biosciences in the country, starting in 2020; this program comprises three main fields of concentration: Parasitology, Microbiology, and Neurosciences [2]. The program currently enrolls 48 students. In 2017, MIHER also supported the implementation of a Ph.D. program in Biosciences and Public Health [2]. Of the 42 students enrolled to date in this Ph.D. program, 40% were directly sponsored by MIHER‑generated grants. So far, three students have successfully completed their Ph.D. degrees, with dissertation research of the remaining students still ongoing.

In 2023, MIHER supported the design of another master’s program in Health Sciences at UniLicungo; this program includes three areas of concentration: Clinical Investigation, Tropical Medicine and Climate Changes, and Health Professionals Education. The first cohort of students (60) was expected to enroll in September 2024.

These postgraduate programs also led to more than 300 micro‑research projects for students, lecturers, and researchers, who were mentored in multidisciplinary areas across communicable and non‑communicable diseases and health professionals education [2, 14–25]. More than 60 student and faculty micro‑research grants were also provided to lecturers via their participating institutions [2].

### Short courses developed and number of attendees

Table 1 summarizes the number and type of research and development (R&D) short courses developed and their participants. During the reporting period, MIHER designed and implemented 260 short courses, which enrolled 9,514 participants. The bioethics and responsible conduct of research (RCR) courses (101) attracted the highest numbers of attendees (5,804 participants).

**Table 1:** Type and number of short courses designed and implemented and number of participants.

YEAR	NO. OF COURSES AND PARTICIPANTS	RESEARCH METHODS*	RCR AND BIOETHICS	GRANT WRITING	MANUSCRIPT WRITING	TOTAL	AVERAGE NUMBER OF PARTICIPANTS PER COURSE
**2011**	No. of courses	1	0	0	1	**2**	**22**
	No. of participants	22	0	0	22	**44**	
**2012**	No. of courses	5	1	0	2	**8**	**21**
	No. of participants	97	36	0	37	**170**	
**2013**	No. of courses	3	0	1	0	**4**	**31**
	No. of participants	92	0	30	0	**122**	
**2014**	No. of courses	2	3	2	0	**7**	**27**
	No. of participants	42	81	69	0	**192**	
**2015**	No. of courses	12	8	4	5	**29**	**24**
	No. of participants	274	171	134	107	**686**	
**2016**	No. of courses	6	6	1	2	**15**	**13**
	No. of participants	118	19	20	43	**200**	
**2017**	No. of courses	14	6	3	4	**27**	**15**
	No. of participants	225	62	40	73	**400**	
**2018**	No. of courses	22	6	4	6	**38**	**17**
	No. of participants	396	80	60	105	**641**	
**2019**	No. of courses	19	27	0	4	**50**	**15**
	No. of participants	154	498	0	78	**730**	
**2020**	No. of courses	11	23	0	1	**35**	**33**
	No. of participants	508	567	0	79	**1154**	
**2021**	No. of courses	2	12	0	2	**16**	**106**
	No. of participants	123	1456	0	123	**1702**	
**2022**	No. of courses	6	9	3	3	**21**	**156**
	No. of participants	221	2834	45	176	**3276**	
**2023**	No. of courses	5	0	2	1	**8**	**25**
	No. of participants	126	0	25	46	**197**	
**Total 2011–2023**	No. of courses	108	101	20	31	**260**	**37**
	No. of participants	2398	5804	423	889	**9514**	

*Notes:* No., number; RCR, responsible conduct of research.

*Research methods courses include biostatistics, epidemiology, clinical research enhancement through supplemental training (CREST), good clinical practice, good laboratory practice, data analysis, and presentation.

Curiously, average participation rates were highest during the coronavirus disease 2019 (COVID‑19) pandemic years (2020–2022), probably because the adoption of distance learning facilitated students’ attendance.

### Research and training grants: applications and management

MIHER supported the writing and submission of 116 grants, of which 98 (84%) were awarded, totaling US $29,940,086. This represents an extraordinary success rate. By way of comparison, the most recent success rate (Fiscal Year 2023) for individual research project grants awarded by the US National Institute for Allergy and Infectious Diseases (NIAID) is only 17% (https://report.nih.gov/funding/nih-budget-and-spending-data-past-fiscal-years/success-rates; accessed October 25, 2024). Of the support facilitated by MIHER, $20,067,045 (67%) was generated through the UEM–UCSD partnership. Of the $24,630,770 in US federal funds, $19,193,807 was awarded directly to MIHER, and $5,436,963 was in the form of sub‑awards. Going forward, MIHER has secured $2,614,914 for research and training activities through 2027 from 13 sources (Supplemental File 1). These grants involved, in total, 27 Mozambican researchers as principal investigators and co‑investigators (an average of 3.4 grants/researcher) whose primary institutions were UEM (8), MCH (12), MOH(3), INS (2), and CSO (2) in partnership with 34 researchers from various partner universities in the USA, Europe, Africa, and Oceania. Eight principal investigators and co‑investigators had a dual affiliation with UEM and MCH.

**Figure 3:**
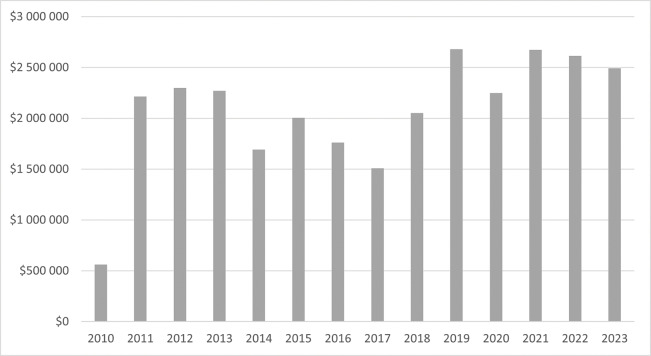
Annual budget of funds administered by MIHER (in USD).

Figure 3 presents the annual amount of funding administered by MIHER.

**Figure 4:**
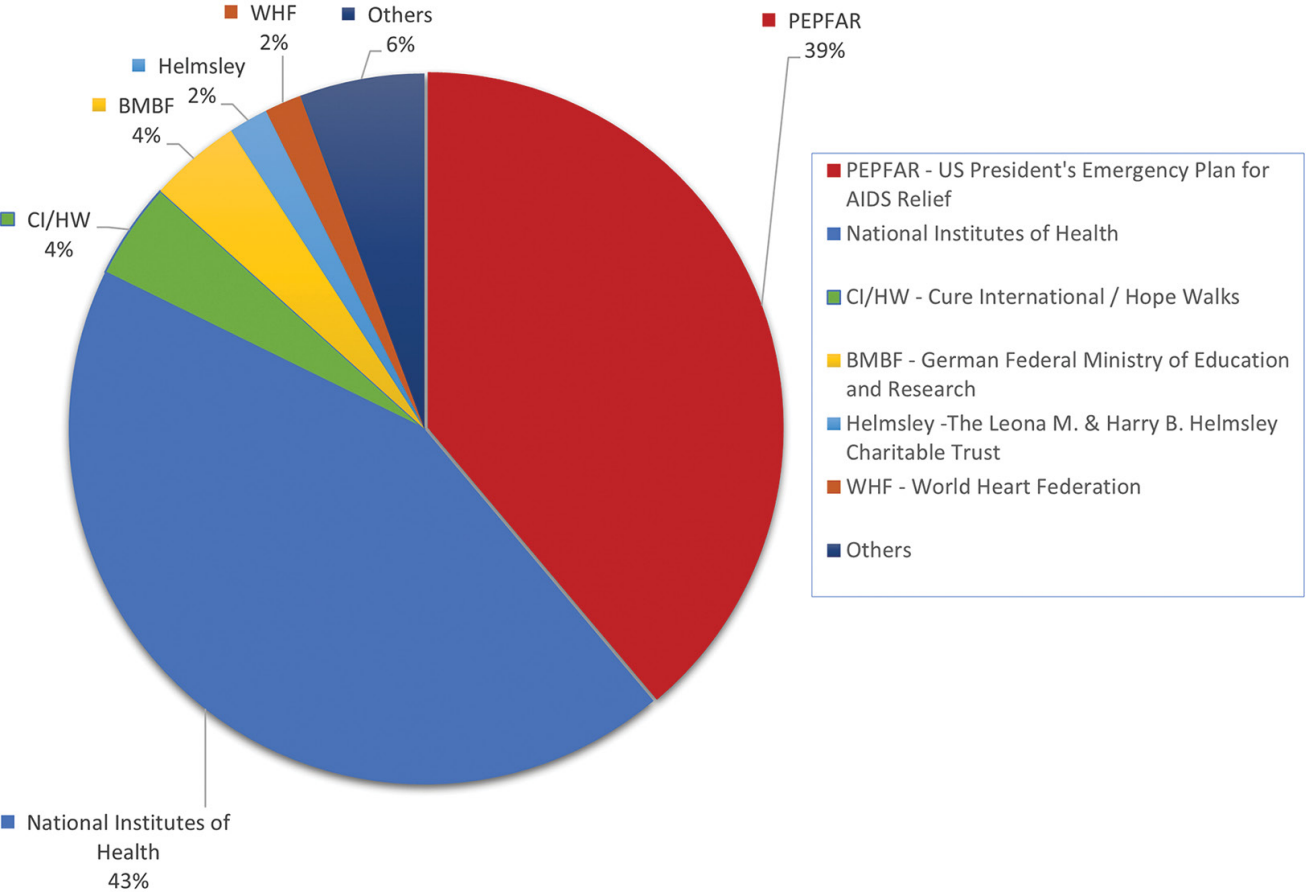
Proportion of grants awarded to MIHER by funding organization.

Figure 4 illustrates the proportion of grants awarded to MIHER by various funding organizations. The major funders have been PEPFAR (39%) and NIH (43%). In terms of geographic representation by continent, most funds came from North America ($27,449,734; 91.7%), followed by Europe ($2,316,504; 7.7%), Oceania ($54,150; 10.2%), and, lastly, Africa ($119,699; 0.4%).

Table 2 presents detailed information of the awards generated through the UEM–UCSD partnership, totaling $20,067,045. Of these funds, 58% came from PEPFAR, and 40.5% came from NIH–FIC. There were also 19 pilot grants for early‑career investigators from the UCSD Center for AIDS Research (CFAR), totaling $592,526.

**Table 2:** Summary information of awards by funding agency, purpose, and amount generated through the UEM–UCSD partnership.

TOTAL FUNDING UEM–UCSD PARTNERSHIP IN USD = $20,067,045			
AWARD DESCRIPTION	FUNDING AGENCY	PURPOSE	AMOUNT IN US DOLLARS, *N* (%)
**Global health**	University of California	Purchase of apartment for visitors	50,000 (0.25)
**MEPI and HEPI**	PEPFAR	Programmatic activities for research, training, and networking	11,645,174 (58.03)
	NIH‑FIC		2,787,136 (13.89)
**Research training D43**	NIH‑FIC	Research training grants	4,420,000 (22)
**UCSD‑CFAR**	NIH‑NIAID	Pilot grants for early‑career investigators	592,526 (3)
**Research development grants**	NIH‑NIAID	Research grants	331,746 (1.65)
**UEM–UCSD**	Gilead Foundation	Bilateral exchange of residents and laboratory enhancements	78,710 (0.39)
**Research development grants**	Stadema Foundation and the World Federation of Hemophilia	Research development	135,176 (0.67)
**Others**	UCSD	Travel support	26,577 (0.14)

MIHER trained 16 research administrators by providing professional development opportunities, such as participation in international networking events, including both in‑person and online conferences organized by the NIH along with the International Society of Research Administrators. MIHER also trained 30 lecturers and researchers from UEM and UniLurio in principles of research administration.

### Scientific publications

Research work mentored and/or supported through MIHER’s grants resulted in the publication of 170 articles, of which 151 (89%) were in journals indexed in PubMed (see Supplemental File 2 for details). The publications were on diverse topics, including communicable diseases (e.g., HIV and its co‑morbidities, tuberculosis, hepatitis, neglected tropical diseases, COVID‑19), non‑communicable diseases (e.g., stroke and mental health), antimicrobial resistance, health professionals education, health system strengthening, nutrition and food security, ethnobotanies, and digital technologies.

**Figure 5:**
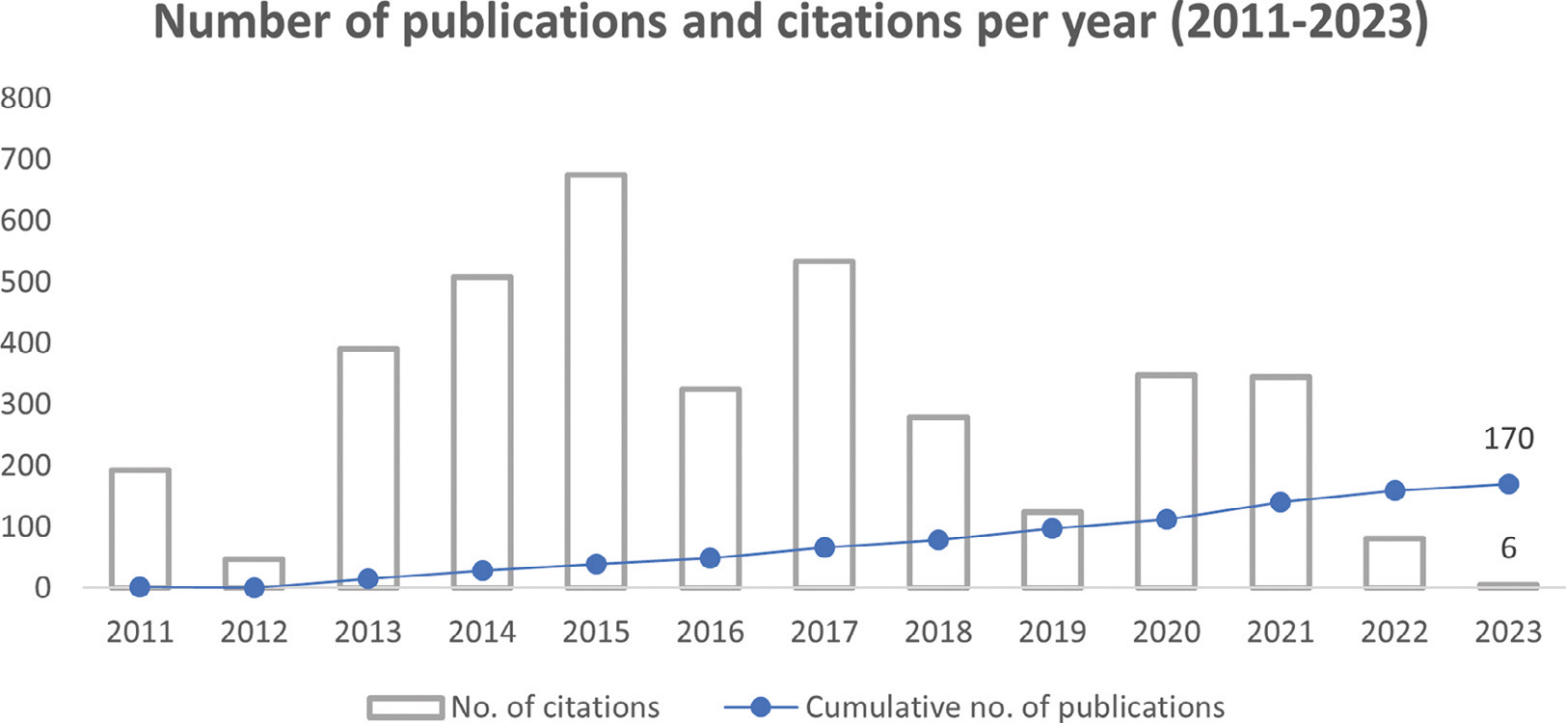
Publication and citation trends of MIHER during the reporting period. Over the years, there has been a steady increase in publication output.

Figure 5 presents a graphical representation of the number of publications and citations per year. Over the years, there has been a steady increase in the publication output of about 13 per year. In December 2023, MIHER’s indexed publications had accumulated 3,860 citations. Out of 170, 96 (57%) manuscripts were published in high‑citation potential journals, while 52 (31%) manuscripts had a lower SJR rank (< 1.0).

An important goal of MIHER was to facilitate domestic research outputs that describe findings from projects completed in Africa (and Mozambique specifically). In fact, consistent with this, Africans served as first or last author for 55% and 34% of the publications, respectively. Of these, 44% had a Mozambican first author. Last authors were most often from the USA (46%), followed by Africans (34%), of whom 23% were Mozambicans. Females were the majority of first authors (58.2%) and served as last authors of 46.4% of publications. Africans outside Mozambique, representing 24 countries, collaborated on 42 publications (see Supplemental Table 1 for details).

### Development of research infrastructure

Investments made in infrastructure to support development of research included the rehabilitation or acquisition of research equipment for the Parasitology and Pathology laboratories of the UEM Faculty of Medicine (FoM‑UEM). Advanced training was also provided by laboratories at UCSD and South Africa [8] to three laboratory technicians and three lecturers on topics such as molecular biology, bioinformatics, immunohistochemistry, cell culture, cultivation of parasites, and techniques to assess microbial resistance.

ICT rooms were installed at FoM‑UEM, MCH, UniLurio [2, 4, 26], and the Instituto de Ciências de Saúde de Quelimane. In addition, MIHER supported the installation of a SEACOM fiber optic terminal at UEM, the extension of internet connectivity to the FoM‑UEM and MCH, and an internet connection at UniLurio [2, 4]. MIHER´s projects and fees collected from master’s students supported the installation of a Center for Graduate Studies at UniLurio. Fees from students also helped to pay supplements to lecturers and administrative staff, as well as other institutional expenses [2]. MIHER also supported the development of an Institutional Bioethics Committee at the FoM‑UEM and MCH [8].

### Communities of practice and organization of scientific events

MIHER participated in the creation of AFREhealth in 2016, a pan‑African community of practice established to enhance training and research capacity development for HCW in the African continent [27]. MIHER representatives occupy 2 of the 23 available seats within the AFREhealth Governing structure.

MIHER has also played a critical role in organizing 11 international scientific events, including the IV MEPI annual Symposium and the VI Annual AFREhealth symposium, held in August 2014 and 2023, respectively, in Maputo. The VI Annual AFREhealth symposium had 500 participants from 131 universities. Speakers from sub‑Saharan institutions (184) were from 23 countries that were Anglophone (71.2%), Lusophone (16.3%), and Francophone (11.4%). Mozambique had the highest number of abstracts submitted (34), followed by Uganda (31) and Zambia and South Africa (17 each). National scientific events at the UEM (1), FoM‑UEM (3), and ISCISA (5) were also supported by MIHER.

## Discussion and Conclusions

MIHER, the first RSC of its kind in Mozambique, has very effectively convened national and international universities, research institutions, and individual researchers with similar needs, but at different stages of development, to jointly share human, financial, and other resources to transform the HRW and research landscape in Mozambique and across the African continent, in a sustainable way.

Hurdles faced by Mozambican researchers in grant writing, submission, administrative and fiscal management, and contracts are being attenuated through MIHER, as an institution open to the entire Mozambican research community [8, 28].

There are similar initiatives being undertaken by other MEPI partners. Some RSCs were created as integral parts of one university (e.g., Kwame Nkrumah University of Science and Technology in Ghana). Others were established at either Faculty of Health Sciences or College of Medicine levels (e.g., University of Nairobi in Kenya). Some RSCs formed consortia to develop research policies, strengthen research management offices, develop research administration curricula, and enhance awareness about the role and relevance of research administration in other universities (e.g., the Zimbabwe Initiative on Research and Innovation Management, composed of the University of Zimbabwe, the Africa University, and the National University of Science and Technology with support of their US partners, Stanford University and the University of Colorado, Denver) [9, 27, 29–31].

MIHER adopted a different model, creating a para‑university not‑for‑profit organization, thus more broadly serving universities, research institutions, researchers, and lecturers across the country, regardless of their public or private status [2, 8].

Regardless of their structure, all RSCs have reported positive outcomes, such as increasing the number of grant applications and their success rate, the number of publications in peer‑reviewed international journals, the number of postgraduate students enrolled in master’s and Ph.D. degree programs, and increased faculty satisfaction, as well as ownership and independence of research efforts [9, 11, 27, 29, 31].

The options offered to students enrolled in master’s and Ph.D. programs to pursue research development and online training fostered improvements in career development opportunities. They also reduced inequities imposed by development imbalances among different regions of a given country. This has also improved faculty retention in regional settings that might otherwise struggle to retain highly trained staff. It has also reduced the strain of supporting faculty if local resources are limited, and leverages support from foreign academic institutions [2, 4, 26].

The research culture as well as the self‑esteem and confidence of Mozambican lecturers and researchers involved in this initiative were substantially increased by MIHER. Lecturers and researchers were highly exposed and interconnected with diverse funding agencies across multiple countries and continents [2, 8, 28]. Indeed, MIHER played a crucial role in academic career development. At the FoM‑UEM, for example, a participant attained the rank of full professor for the first time since the FoM‑UEM was established, in part because of this support; many others have now followed.

Funds acquired through research grants improved the satisfaction of faculty members, researchers, and other HCW, and created an adequate research environment fostering collaboration and retention of better‑qualified human resources within institutional settings.

Research institutions in Mozambique are extremely dependent on external funding for research activities. Indeed, most research funding (more than 80%) had been acquired through bilateral collaborations between the Mozambican Government and others, including the USA, Sweden, Italy, and Norway [28, 32]. While these earlier funding streams played an important role in institutional research development, they did not necessarily stimulate local researchers to search actively for additional competitive funding opportunities and to establish strong international collaborations that would favor a true sustainable nationwide research, development, and training network, as now intended by MIHER.

The sustained increase in publications (96% in PubMed indexed journals) and grants awarded, over more than a decade, illustrates the positive impact of MIHER’s endeavors on the research and development capacity of its institutional and/or individual partners. Indeed, prior to the inception of MIHER, our investigators had little if any understanding of the availability of research grants, especially from international sources such as the US federal government, and thus, support for research was sporadic at best. In addition, the administrative support provided by MIHER allowed our investigators to be extraordinarily successful in applying for grants once they were aware of these opportunities. Furthermore, scientific knowledge contributes importantly to public health awareness, and will help to break (institutional, political, and/or religious) barriers that otherwise would attenuate progress in biomedical sciences in a country such as Mozambique with many other apparent challenges and priorities. Healthcare improvements via research translation may have unanticipated positive impacts on the well‑being of populations, while also providing evidence‑based decision‑making for both clinical practice and policymaking [2, 15–17, 23, 33].

Though Africa is home to 25% of the world’s population and 25% of the global disease burden, it participates in only around 2% of the world’s publication output, and most of these papers are principally authored (first and last authors) by scientists from high‑income countries [8, 32, 34]. Interestingly, the MIHER initiative reversed this trend; first authors of most of MIHER’s publications are African, being Mozambican in nearly 80% of these cases. These numbers evolved positively from a previous report published by our group that focused on publications from 2001 to 2010; during that period, only 29% of the 202 peer‑reviewed publications mentioning FoM‑UEM faculty and/or MCH physicians had Mozambican first authors [8]. Similar trends over time are seen for last authors from Mozambique and Africa, with MIHER again associated with a salutary impact. The data suggest that it is possible to develop a research culture where Africans/Mozambicans can not only increase their engagement with research studies but also lead them.

An examination of publications supported by MIHER from 2011 to 2023 also showed increased numbers of African co‑authorships; 25% of the publications included authors from two or more members of the 24 reported African countries. This contrasts with a study published by the Southern African Development Community (SADC) reporting that only 5% of SADC papers published between 2005 and 2008 involved co‑authorships of SADC researchers with other African researchers [32].

MIHER’s initiative also attenuated global gender (male > female) inequities observed for research publications in the Health Sciences. In our series, women frequently served as first or last authors of peer‑reviewed publications, demonstrating an active involvement of Mozambican women in science.

It is undeniable that, if research leadership, ownership, and other resources are made available and funding agencies flexibly support not only north–south but also south–south collaborations (as seen with the PEPFAR and NIH‑FIC initiatives), collaborative research has a greater likelihood of success and impact [35]. Initiatives to avoid research fragmentation and increase collaboration among African countries might enhance sustainability and accelerate the adoption of best practices in scientific research and training. For instance, through AFREhealth, key researchers across Africa have jointly outlined the need for research‑oriented capacity‑building and scientific development in sub‑Saharan African countries to ensure effective and accountable responses to future disease pandemics, both communicable and non‑communicable [36]. As illustrated in this case report, the current situation of low scientific productivity by African researchers can be reversed, provided there is adequate funding support from international partners and (trans)nationwide initiatives (such as MIHER) affording support, management, and dissemination of research and training opportunities in African countries [9, 37, 38].

Initiatives (other than MEPI) intended to strengthen sub‑Saharan African research capacity development include the Africa Research Initiative and Support (ARISE) consortium, composed of universities in Malawi, Rwanda, Zimbabwe, and Uganda, and funded by the Ministry of Foreign affairs of the Netherlands. This effort aims to develop and consolidate a network of RSCs within the consortium in the field of poverty‑related diseases (HIV, tuberculosis [TB], and malaria) by providing training and courses (conduct of clinical research, biostatistics, evidence‑based medicine, data, and grants management), provision of services (epidemiological and statistical support, and data and grant management support and monitoring), and governance and infrastructure (establishing themselves in the center of the research activities in their respective host institutes). They have made vital contributions to the development of research policies and guidelines), and it is expected that, with this initiative, a shift of research ownership will occur such that projects will be led by local institutions [11].

Another example includes the four networks of excellence (NoE) supported by different generations of the European Union‑funded European and Developing Countries Clinical Trials Partnership (EDCTP) [39].

Initiatives with similar approaches, including the World Health Organization (WHO)/Tropical Disease Research (TDR), the Wellcome Trust, the Better Health Information for Better Health Policy (INDEPTH) network, the Consortium for Health Systems Policy Analysis in Africa (CHEPSAA), and the partnership between Makerere University and Karolinska Institutet have reported equally positive outcomes [32, 37, 38, 40].

In all of these initiatives, leadership, ownership, joint planning, alignment with local priorities, and funding flexibility were critical contributors to success [32, 35, 37, 38, 40].

Scientific research and training initiatives must synergize with other programs designed for African developing countries to hasten the spread of best decision‑making and healthcare practices. The training of HCW (300) for the treatment of epilepsy (more than 10% prevalence) in Zambezia province is a fine example of this situation [15]. Including research topics in health professionals’ education in parallel with the use of ICT methodologies (such as tablets, mobile devices, and information about open access technology resources) should become a standard practice for quality improvement and integration of advanced methods into daily‑basis healthcare routines [18, 41]. For instance, technology transfer, including the use of fine needle aspiration cytology for the diagnosis of infectious diseases [33], development of point‑of‑care diagnostics [42], and training in molecular diagnostic techniques [22], were critical to improve research capabilities from epidemiological to fundamental sciences. These technologies are increasingly promoting innovation in clinical practice.

Supporting the establishment of an Institutional Bioethics Committee [8] underscores MIHER’s commitment to upholding ethical considerations in research, thus ensuring timely initiation of human research protocols and protection of research participants. The rehabilitation of research infrastructures (e.g., laboratories and equipment set‑ups), access to virtual libraries and e‑resources, and improvements in internet connectivity all supported research development and further augmented access to digital resources, while creating adequate learning and mentoring environments [8, 28].

MIHER’s networking has facilitated collaborative research among Mozambican and African universities, as well as with universities from high‑income countries, including the USA, Brazil, and Australia and those in Europe and Asia [2, 8, 30], in addition to bringing valuable expertise and resources to Mozambique. The efforts to promote south–south and north–south collaborations facilitated new joint grant applications, academic exchanges, and access to cutting‑edge methodologies/technologies, thus promoting Mozambique’s visibility in the global research community. The successful co‑organization of the 4th MEPI annual symposium and 6th AFREhealth annual symposium in Maputo are clear examples of these.

### Challenges, lessons learned, and the way to move forward

Dealing with multiple funding agencies, with different research infrastructures and with particularities concerning grant requirements and fiscal management, has been a major challenge, mostly because these requirements are far from standard Mozambican national operating procedures. In general, solutions acceptable for both parties emerged from open dialogue and thorough discussions.

Without question, the training opportunities inherent in MIHER enabled more researchers to apply for advanced (MSc and Ph.D.) studies, while feeling confident to pursue parallel research careers. In this context, both MIHER and other Mozambican universities and research institutions now face a new challenge in dealing with a shortage of qualified mentors and research funds to meet the training needs of the continuously increasing number of early‑career researchers. A focus on increasing the development of mentors should be an important follow‑up priority.

Intense competition for international grants, combined with financial constraints faced by funding agencies in their own countries, may undermine prospects for raising research funds. Mozambique’s own internal threats, such as terrorism in the north and the increasing number of extreme climate events, including cyclones and floods [1, 43, 44], make it difficult for the public sector to consistently fund research and training activities. This situation may also negatively affect MIHER’s efforts to build and consolidate an advanced research network in the country.

The lessons one can take so far are that MIHER’s continuous efforts, strategic vision, mission, perseverance, and funding diversification have paid off and should be prioritized.

In summary, the foundation of MIHER contributed to a unique pillar of research and training sustainability, which was built on a network platform involving national and international research and funding partnerships. As such, RSC’s of Excellence, such as MIHER, should be replicated in Mozambique and elsewhere. By nurturing local talent; fostering resource sharing, partnerships, and collaborations; and advocating for research‑driven policies, these centers can further contribute to the development of robust research ecosystems, a motivated HRW, improved healthcare systems, and ultimately, better health outcomes for the global population.

MIHER has enabled local academic capacity to make significant contributions, which will dramatically change the RHW and the research landscape in Mozambique and ultimately in Africa. Above all, these achievements were possible because of the scientific engagement and vision of our collaborators, from UCSD and elsewhere, who value shared goals, mutual respect, coordination, and transparency as critical elements of success in global health research [35, 37]. We are also grateful for similar reasons to our primary funding agencies, PEPFAR and NIH‑FIC. Both entities supported south–south collaboration and leadership and ownership by Mozambicans and Africans to define activities according to their priorities and context.

Moving forward, MIHER aspires to establish itself as a regional research administration training hub especially to serve the Lusophone community in Africa, Latin America, and Asia.

## Data Availability

The datasets generated and/or analyzed during current study are available in supplementary files and or upon request.

## Additional Files

The additional file for this article can be found as follows:

Supplementary material Table 1. Authorship position and distribution by country and by continent.

Supplementary material File 1. Supplemental file 1, List of research grants written and awarded.

Supplementary material File 2. List of publications generated in the reporting period.
