# Supplementary material for: Transforming the Health Research Workforce in Mozambique: Achievements of the Mozambique Institute for Health Education and Research (MIHER) over a 13‑Year Journey
Source: Ann Glob Health. 2024 Dec 9;90(1):78. doi: 10.5334/aogh.4528 (PMC11639690; doi:10.5334/aogh.4528)
Supplement: Supplementary material Table 1. — Authorship position and distribution by country and by continent. [file agh-90-1-4528-s1.pdf]

Table 3. Authorship position and distribution by country and by continent.

Continent	Country	1st author N=170(%)	2nd author N=159(%)	3rd author N=149(%)	Last author N=166(%)
**Africa**	Cameroon	2 (1.2)	3 (1.9)	2 (1.3)	-
	Congo	-	-	-	1 (0.6)
	DR Congo	-	-	1 (0.7)	-
	Egypt	-	-	1 (0.7)	1 (0.6)
	Ethiopia	1 (0.6)	-	-	1 (0.6)
	Ghana	1 (0.6)	-	2 (1.3)	-
	Kenya	-	-	-	1 (0.6)
	Mozambique	74 (43.5)	71 (44.7)	69 (46.3)	39 (23.5)
	Nigeria	2 (1.2)	4 (2.5)	3 (2.0)	-
	Senegal	-	1 (0.6)	-	-
	South Africa	9 (5.3)	6 (3.8)	11 (27.5)	14 (8.4)
	Uganda	4 (2.4)	5 (3.1)	1 (0.7)	-
	Zambia	1 (0.6)	-	-	-
	**Total**	94 (55.3)	90 (56.6)	90 (60.4)	57 (34.3)
**Asia**	Afghanistan	-	-	1 (0.7)	-
	China	-	1 (0.6)	1 (0.7)	1 (0.6)
	India	-	-	2 (1.3)	-
	**Total**	-	1 (0.6)	4 (2.7)	1 (0.6)
**Europe**	Belgium	-	1 (0.6)	-	1 (0.6)
	Denmark	-	1 (0.6)	-	2 (1.2)
	France	1 (0.6)	-	1 (0.7)	3 (1.8)
	Germany	-	1 (0.6)	-	1 (0.6)
	Italy	1 (0.6)	-	-	-
	Ireland	-	-	1 (0.7)	-
	Norway	1 (0.6)	1 (0.6)	1 (0.7	-
	Netherlands	-	-	1 (0.7)	-
	Portugal	4 (2.4)	6 (3.8)	2 (1.3)	10 (6.0)
	Spain	-	-	1 (0.7)	2 (1.2)
	Sweden	-	-	-	3 (1.8)
	Switzerland	1 (0.6)	1 (0.6)	-	1 (0.6)
	Uk	2 (1.2)	2 (1.3)	1 (0.7)	1 (0.6)
	**Total**	10 (5.9)	13 (8.2)	8 (5.4)	24 (14.5)
**Oceania**	Australia	3 (1.8)	-	1 (0.7)	6 (3.6)
	New Zealand	-	-	1 0.7)	-
	**Total**	3 (1.8)	0	2 (1.3)	6 (3.6)
**America**	Peru	-	-	1 (0.7)	-
	Brazil	-	-	1 (0.7)	2 (1.2)
	Nicaragua	-	1 (0.6)	-	-
	US	63 (37.1)	54 (34.0)	43 (28.9)	76 (45.8)
	**Total**	63 (37.1)	55 (34.6)	45 (30.2)	78 (47.0)
